# 

**Published:** 1866-01

**Authors:** 


### The Chicago Medical Journal.

A MONTHLY JOURNAL

DEVOTED TO THE CULTURE OF MEDICAL AND SURGICAL SCIENCE.

It will be our aim to secure for our readers original papers on important medical subjects; to present valuable selections from foreign journals, and from our large exchange list of American medical periodicals; to obtain reports of interesting cases, observed in the Hospitals, Infirmaries and Dispensaries of Chicago, and to call the attention of the Profession to new and valuable works in the various departments of medical science.

While it will be our chief object to aid in promoting the best interests of the Profession in the culture of true science, we shall also carefully endeavor to publish such intelligence, not strictly scientific, as may be of particular interest to the practising physicians of the Northwest.

All letters for the Journal should be addressed to R. M. Lackey, M.D., P. O. Box 2175, Chicago, Ill. *Office—169 South Dearborn Street.*

TERMS—$2 PER ANNUM, IN ADVANCE.

SINGLE COPIES, 25 CENTS.

